# Neuroimmunology Research. A Report from the Cuban Network of Neuroimmunology

**DOI:** 10.3390/bs8050047

**Published:** 2018-05-08

**Authors:** María de los Angeles Robinson-Agramonte, Lourdes Lorigados Pedre, Orlando Ramón Serrano-Barrera

**Affiliations:** 1 Neuroimmunology Laboratory, Immunochemical Department, International Center for Neurological Restoration, Ave 25 # 15805 b/w 158 and 160, Playa, Havana 11300, Cuba; lourdesl@neuro.ciren.cu; 2 Las Tunas General Hospital, Post Office Box 27, Las Tunas 75100, Cuba; orlandosb@infomed.sld.cu

**Keywords:** neuroimmunology, neurodevelopmental disorders, neurodegenerative disorders, non-invasive brain stimulation, Alzheimer disease’s, Parkinson’s disease, autism, demyelinating disease, neuropsychology, ataxy

## Abstract

Neuroimmunology can be traced back to the XIX century through the descriptions of some of the disease’s models (e.g., multiple sclerosis and Guillain Barret syndrome, amongst others). The diagnostic tools are based in the cerebrospinal fluid (CSF) analysis developed by Quincke or in the development of neuroimmunotherapy with the earlier expression in Pasteur’s vaccine for rabies. Nevertheless, this field, which began to become delineated as an independent research area in the 1940s, has evolved as an innovative and integrative field at the shared edges of neurosciences, immunology, and related clinical and research areas, which are currently becoming a major concern for neuroscience and indeed for all of the scientific community linked to it. The workshop focused on several topics: (1) the molecular mechanisms of immunoregulation in health and neurological diseases, (like multiple sclerosis, autism, ataxias, epilepsy, Alzheimer and Parkinson’s disease); (2) the use of animal models for neurodegenerative diseases (ataxia, fronto-temporal dementia/amyotrophic lateral sclerosis, ataxia-telangiectasia); (3) the results of new interventional technologies in neurology, with a special interest in the implementation of surgical techniques and the management of drug-resistant temporal lobe epilepsy; (4) the use of non-invasive brain stimulation in neurodevelopmental disorders; as well as (5) the efficacy of neuroprotective molecules in neurodegenerative diseases. This paper summarizes the highlights of the symposium.

## 1. Introduction

Neuroimmunology has arisen as an innovative and integrative field in the shared edges of neurosciences, immunology, as well as related areas. The current research was carried out in order to get insight into the current development of neuroimmunology, in terms of scientists, events and literature shaping and reflecting its main achievements: the delineation and evolution of a central paradigm. A first topic addressed was to review some events on the history of neuroimmunology. This talk fixed the roots of neuroimmunology in the 19th century, with the descriptions of some of the model diseases (e.g., multiple sclerosis, neuromyelitisoptica, Guillain-Barré syndrome), the diagnostic examination of cerebrospinal fluid (CSF) by Quincke or the development of neuroimmunotherapy with the vaccine for rabies by Pasteur, and it showed that the field began to become delineated as an independent research area in the 1940s, after Medawar’s statements on the peculiarities of the brain in antigenic tolerance and the findings by Waksman and Adams on experimental allergic (later coined as autoimmune) encephalomyelitis. Reviewed aspects of neuroimmunology were shown to have evolved from neurological infections and immunopathological conditions to the conformation of a supracontrol system integrating neural and immune mechanisms toward a better adaptation of the organism in an ever-changing environment, and this allowed for a definition of the purpose of the work and its significance in this field [1,2].

This meeting was the fourth edition of the International Workshop of Neuroimmunology and the first of the Cuban Network of Neuroimmunology “CUBANNI”, going from the 10 to 14 June 2017. The first session chaired by Professor Von Wee Yong and Professor Maria Robinson opened with an atypical but very interesting talk from Prof. Orlando Serrano from Tunas University, Cuba. This talk offered an overview on the history of neuroimmunology. After that, some very experienced speakers in this field showed some advances in this field and discussed current topics relative to how the immune reaction occurs in the context of the neurological disorders, as well as discussing, in some cases, ways to restore functions or to modulate aberrant mechanisms to get beneficial effects that are disease related. Several diseases were revised, such as multiple sclerosis (MS), Alzheimer’s disease, epilepsy, strock, autism, Parkinson disease, as well as others. Interesting lectures served as guides to offer a better understanding of the molecular processes underlying neuroimmune pathology in neurodegenerative, neurodevelopmental, and neurovascular diseases, as well as to understand some vulnerabilities and comorbidities occurring in these disorders. Relevant to this topic was the lecture of Prof. Jack Antel on a new approach in microRNA in MS vulnerabilities [1].

The papers that referred to new therapeutic approaches for interventions in neuroimmunology were presented by experts: Prof. Diogo O Souza from UFRGS, Brazil; Prof. Von Wee Yong from the University of Calgary, Canada, Prof. Luis Velazquez from the Center for the Research and Rehabilitation of Hereditary Ataxias (CIRAH), Holguín, Cuba, Prof. Lázaro Gómez from the International Center for Neurological Restoration (CIREN), Havana, Cuba, and Prof. Miriam Marañón Cardonne from the Applied Electromagnetism Center, Santiago de Cuba, Cuba. An experiment in the use of protector molecules in neurological diseases was also presented by Prof. Yanier Núñez-Figueredo from the Center for Drugs Research and Development (CIDEM), Havana City, Cuba.

## 2. Lecture Highlights

Manfred Schedlowski, from the Institute of Medical Psychology and Behavioral Immunobiology, University Clinic Essen, Germany, delivered his lecture entitled “Learned immune responses—mechanisms and potential clinical application”.

This speaker introduced experimental data in rodent and humans demonstrating that peripheral humoral and cellular immune functions can be modulated by associative learning processes. In a taste-immune learning paradigm in rats employing the immunosuppressive drug cyclosporine A as an unconditioned stimulus, the behaviorally conditioned suppressive effects on IL-2 and IFN-γ mRNA expression, cytokine production and T cell activity were shown to be centrally mediated via the insular cortex and amygdala, and peripherally via the sympathetic innervation of lymphatic organs and the beta-adrenoreceptor-dependent inhibition of the calcineurin activity. This learned immunosuppression is of biological relevance, since behavioral conditioning significantly attenuated allergic responses and symptom severity in chronic inflammatory autoimmune diseases. Moreover, behaviorally conditioned immunosuppression has also been demonstrated in healthy humans and in diseases. The presented data provided proof of the principle that learning strategies can be implemented in a immunopharmacological regimen, the aim being to reduce the amount of drugs, thereby minimizing unwanted toxic drug side effects while maximizing the therapeutic benefit for patients [3,4].

Professor Wekerle from the Max Planx Institute, Germany entitled his lecture: “Nature and Nurture-Gut Microbiota and Multiple Sclerosis”. This very experienced investigator commented on his work group’s extensive experience, spanning multiple years, in using a spontaneous variant of relapsing-remitting experimental autoimmune encephalomyelitis (EAE) to study the triggering events of brain autoimmunity. Single transgenic JL/J mice expressing a myelin oligodendrocyte glycoprotein (MOG) showed autoreactive T cell receptor in about 70% of their CD4+ T cell repertoire. He showed how the disease risk is modulated by the modification of the microbiota, for instance by antibiotic treatments or by dietary interventions, as well as showing how they currently explore the effect of microbial samples from people with MS on initiation of EAE in the RR-mouse model. Prof. Wekerle’s pioneering work in this field also exposed the previous results observed in a rodent model with spontaneous EAE, establishing the relevance of gut flora in disease-triggering demyelination [5,6,7,8]: gut-associated lymphoid tissue, gut microbes and the susceptibility to experimental autoimmune encephalomyelitis [9].

The human gut commensal microbiota forms a complex population of microorganisms that survive by maintaining a symbiotic relationship with the host. Amongst the metabolic benefits, it plays an important role in the formation of an adaptive immune system and in the maintenance of its homeostasis, and it is crucial in the induction, training, and function of the host immune system. An optimal immune system-microbiota alliance allows the induction of protective responses to pathogens and the maintenance of regulatory pathways to the tolerance to innocuous antigens. Nevertheless, a mutual impact of the gastrointestinal tract (GIT) and central nervous system (CNS) functions was recognized since the mid-twentieth century, and it has been accepted that the so-called gut-brain axis provides a two-way homeostatic communication, through immunological, hormonal and neuronal signals [10,11].

The GIT might represent a vulnerable area through which pathogens influence all aspects of physiology, even inducing CNS neuro-inflammation. A dysfunction of this axis has been associated with the pathogenesis of some diseases outside the GIT that have shown an increase in incidence over the last decades. Some papers had looked at how the commensal gut microbiota influences a systemic immune response in some neurological disorders, highlighting its impact cognition in multiple sclerosis, Guillain-Barrè syndrome, neurodevelopmental and behavioral disorders and Alzheimer’s disease [12]. Nevertheless there no full comprehension on the implication of the potential microbiota-gut-brain dialogue in neurodegenerative diseases that might provide an insight on the pathogenesis and therapeutic strategies of these disorders.

The discussion of this talk focused on the fact that genes identified as having a susceptibility to MS do not satisfy the risk that argued for MS development. It was emphasized that many environmental risk factors to MS are described [13,14], but a growing number of studies demonstrate that gut microbes could directly play a role in the onset or progression of MS. A study led by researchers at the Max Planck Institute in Germany and co-authored by Cekanaviciute and Baranzini was referred to, in which it was found that microbiome transplants from MS patients could exacerbate symptoms in mice with a genetic model of the disease and which identified that specific species of bacteria more common in people with MS—*Akkermansia muciniphila* and *Acinetobacter calcoaceticus*—triggered the cells to become pro-inflammatory responses in human peripheral blood mononuclear cells and in monocolonized mice, while a species of bacteria found at lever that were lower than usual in MS patients—*Parabacteroides distasonis*—triggered immune-regulatory responses, stimulating anti-inflammatory IL-10-expressing human CD4^+^CD25^+^ T cells and IL-10^+^FoxP3^+^ Tregs in mice [13,14].

Finally, the experiment with microbiota transplants from MS patients to germ-free mice resulted in more severe symptoms of experimental autoimmune encephalomyelitis and reduced proportions of IL-10+ Tregs compared with mice that were “humanized” with microbiota from healthy controls. This study identified specific human gut bacteria that regulate adaptive autoimmune responses, suggesting a therapeutic targeting of the microbiota as a treatment for MS at time that provided evidence that MS-derived microbiota contain factors that precipitate an MS-like autoimmune disease in a transgenic mouse model.

Microbial colonization of the infant gastrointestinal tract (GIT) begins at birth, is shaped by the maternal microbiota, and is profoundly altered by antibiotictreatment [15,16]. Antibiotictreatment of mothers during pregnancy influences the colonization of the GIT microbiota of their infants [15]. The role of the GIT microbiota in regulating the adaptive immune function against systemic viral infections during infancy remains undefined. Nevertheless, an undisturbed colonization and progression of the GIT microbiota during infancy are necessary to promote robust adaptive immune responses. The topic of symbiotic and antibiotic interactions between gut commensal microbiota and the host immune system was discussed, regarding the integral elements of commensal microbiota that stimulate responses of different parts of the immune system and lead to health or disease, as well as on the conditions and factors that contribute to gut commensal microbiota’s transformation from a symbiotic to an antibiotic relationship with humans [16]. The authors suggested to continue research efforts to better understand the host-microbiota interactions in physiological and disease settings, which might lead to the development of rational-based treatments.

For decades, the brain was considered an autonomous tissue that performs best without any assistance from the immune system. It is now widely accepted, largely through our work, that circulating monocytes and CD4+ T cells are needed for supporting brain reparation and functional plasticity. It was demonstrated that leukocytes could gain access to the brain’s territory through a unique interface, the epithelial layer that forms the blood-CSF-barrier, the choroid plexus (CP) [5,8]. Michal Schwartz and Aleksandra Deczkowska, from The Weizmann Institute of Science, Rehovot, Israel, exposed these considerations in their lecture entitled “Reversing age-related dementia and Alzheimer’s disease by restoring the brain-immune axis”. Evidences from a Robust RNAseq analysis demonstrated that, in aging, the function of this interface is suppressed due to a systemic loss of interferon-gamma (IFN-γ), and due to a local elevation of type I interferon (type 1-IFN), which not only interferes with the activity of the CP but also negatively affects microglial activity. The intracerebral administration of anti-IFN-β receptor antibodies restored CP activity, reversed the microglial cognitive-impairing phenotype, and improved the overall cognitive ability in aged mice [17,18,19,20].

In an animal model of Alzheimer’s disease, the CP was found to be dysfunctional, mainly due to low IFN-γ availability, which results from systemic immune suppression/exhaustion. Transiently reducing the systemic immunosuppression via the depletion of Foxp3+ regulatory T cells, or via the blocking of the inhibitory PD-1/PD-L1 immune checkpoint pathways, led to an increase of the activation of the CP to express trafficking molecules, which in turn led to the recruitment of immune regulatory cells to sites of brain pathology. Treatments with anti-PD-1 antibodies were found to be effective in several mouse models of AD in reversing cognitive loss, the removal of plaques, and in restoring brain homeostasis as determined by the inflammatory molecular profile. Such an approach is not meant to be against any single disease-escalating factor in AD, but rather empowers the individual’s immune system to drive the process of repair. By directly targeting the immune system, rather than a single disease risk factor in the brain, this approach provides a comprehensive therapy that addresses numerous factors that become detrimental in the AD brain [21].

The role of the S100B protein, in neuroinflammation and Alzheimer’s disease, was presented by Prof. Carlos-Alberto Gonçalves from the Federal University of Rio Grande do Sul, Puerto Alegre, Brazil. Alzheimer’s disease (AD) is characterized by two main neuropathological brain patterns: the senile plaques formed by deposits of the β-amyloid protein (Aβ) at cholinergic terminals and the neurofibrillary tangles produced by tau protein hyper-phosphorilation. This evidence points to the fact that AD is not restricted to neurons; that neuroinflammation precedes AD pathogenesis; and that glial activation occurs early on in AD, even before cognitive deficit and amyloid deposition. Like microglia, astrocytes recognize danger-associated and pathogen-associated molecular patterns and release inflammatory mediators, as well as supporting and modulating neuronal activity. S100B is a calcium-binding protein mainly expressed in astrocytes in brain tissue, and experimental evidence indicates its involvement in neuroinflammation and degenerative disorders, including AD. This protein has many putative intracellular targets; it is secreted and has autocrine and paracrine effects on glia, neurons and microglia, while showing a toxic effect at a high concentration [22]. Some proposed intracellular targets of S100B include proteins of the cytoskeleton (e.g., GFAP), modulators of the cell cycle (e.g., p53 and Ndr kinase), the protein kinase C and phosphatase 2B (calcineurin). The extracellular effect of S100B, observed in neural cultures, depends on its concentration, since it is neurotrophic at pico and nanomolar levels and apoptotic at micromolar levels. The activation of RAGE and signaling pathways such as ERK, NF-κB and NFAT, following the effects of S100B observed in culture were discussed by Prof. Gonçalves, along with its role in the inflammatory response and its putative use as a biomarker in the three stages of AD [23,24,25,26,27].

The results of a novel study on the participation of neuroinflammatory processes in neurological pathologies such as epilepsy were shown. In this field, Dr. Lourdes Lorigados and collaborators presented clinical evidence of the involvement of inflammatory processes both centrally and peripherally in drug-resistant epilepsy with their work entitled: Two year follow-up of IL-1, IL-6 in temporal lobe drug-resistant epilepsy patient treated surgically [28,29].

Autism spectrum disorders are neurodevelopmental disorders which involve moderately to severely disrupted functioning in regard to social skills and socialization, expressive and receptive communication, and repetitive or stereotyped behaviors and interests [30]. “How Neuropsychology informs Neuroimmunology—Understanding the Cognitive and Behavioral Vulnerabilities in Autism Spectrum Disorders”, was the lecture delivered by Prof. Scott Hunter of Chicago University, USA. This presentation addressed the current understanding of the neuropsychology of autism spectrum disorders (ASD), in association with ongoing research on neuroimmunological factors that contribute to higher risks of neurodevelopmental disorders. The spectrum of challenges experienced by children with ASD and the multiple levels of influence that occur neurodevelopmentally, both prenatally and postnatally, were reviewed [31]. A proposal for an integrated collaboration between researchers and clinicians was also offered, to guide considerations regarding etiology and treatment in this disorder.

“From clinical to molecular in autism: A pending task to the science”, delivered by Prof. Maria Robinson Agramonte from the International Center for Neurological Restoration, Havana, Cuba, was based on a study involving clinical and molecular analysis. It showed how necessary it became to perform multidisciplinary studies in autism in order to advance the understanding of the disease pathology toward better clinical management and toward the implementation of novel therapies in development. Referring to the contribution of inflammatory markers to vulnerabilities in autism, this paper showed the potentialities of EEG findings in aiding information on clinical decision making for children with autism spectrum disorders [32].

Dalina Laffita from Florida Atlantic University, delivered a talk entitled “Suppressing Igf-1 signaling pathway rescues neuronal overgrowth of Pten+/- mice”. Macrocephaly is associated with ASD. The alteration of mTOR signaling pathways has been shown to be involved in 14% of ASD individuals [33]. The research supports divergent trajectories of brain growth in the regressive vs. non-regressive phenotype of ASD; however, the degree to which the cellular mechanisms underlying these profiles differ is not clear [34]. PTEN is a gene associated with ASD, the macrocephaly is observed in the heterozygous mutation in PTEN (PTEN+/-), which leads to a negative regulation in PI3K/Akt/mTOR signaling pathways; it is a great model for studying ASD, as these mice show social behavioral deficits that are observed in humans. During this study, there was evidence that Pten is a regulator of β-catenin. An increase was seen in the β-catenin signal, but the manner in how both of these are associated is not well understood. Measurements of the soma cell size of the fifth layer of the cerebral cortex in a Pten+/- mouse were taken. There was a decrease in the sizes of the neurons; thus, the ultimate goal was to recover the original size after birth, by upregulating the PI3K/Akt/mTOR signaling pathway. The inhibition of the insulin-like growth factor receptor 1 (IGF1R), which is found upstream of this pathway, led to a significant decrease in soma cell size in Pten+/-/Igf-1r+/- mice. The authors considered this a very promising result because it opens the door for a new pharmacological agent that could recover the damage done by this mutation. The pathway being targeted is a cellular pathway, so this brings up the challenge that the pharmacological agent must be able to cross the blood brain barrier and be able to only influence this cellular pathway within the brain to prevent side effects.

Georg Auburger from Goethe University Hospital Frankfurt, Germany, delivered a lecture on Ataxin-2 as a translation factor with a specificity for mitochondrial precursors. Prof. Auburger introduced his lecture by referencing the Ataxin-2 (ATXN2) gene family characterization, saying that, via yeast, it extends from man to plants and that it appears to be so important for all forms of eukaryotic life that several gene copies exist in most vertebrates, as well as in plants and fungi. Mammalian Ataxin-2 is transcriptionally induced during starvation; he said and continued saying that in periods of nutrient depletion, oxidative stress or infection, its subcellular localization changes from the ribosomal translation apparatus to stress granules, where RNA quality control and RNA repair occur [35].

Ataxin-2 is a key element of the stress granules component. Over the last two years, it was shown in yeast, worms and mammals that Ataxin-2 represses global mRNA translation via diminished mTORC1 signaling, a pathway where several drugs are already available. Importantly however, the abundance of selected factors requires Ataxin-2 expression. One example is the PAS domain containing the factor period. The PAS domains are signal modules that are widely distributed in proteins across all kingdoms of life. They are common in photoreceptors and transcriptional regulators of eukaryotic circadian clocks and mainly possess protein-protein interaction and light-sensing functions [36], which triggers rest and the decline of metabolic activity during nighttime. Another example is the mitochondrial matrix enzyme Iso-Valeryl-Dehydrogenase, which is responsible for leucine degradation and thus reduces mTORC1 activity. Further examples are branched chain amino acid enzymes in the mitochondrial matrix, which are responsible for fatty acid degradation. Additionally, mitochondrial matrix enzymes in the tricarboxylic acid (TCA) cycle of glycolysis are enhanced by Ataxin-2 expression. When Ataxin-2 is not absent, fat accumulates subcutaneously and in liver lipid droplets, parallel to glycogen granules. Thus, the recruitment of stored alternative fuels in times of glucose depletion or of excessive bioenergetic demands is dependent on Ataxin-2. Given that cellular lipid uptake via the endocytosis of trophic receptors is impaired by Ataxin-2, cells are more dependent on their own nutrient reserves, and autophagy would probably increase. Interestingly, Ataxin-2 expression also enhances the abundance of PINK1, an autophagy-regulator that is responsible for the quality control and repair of mitochondria. A conclusion of this lecture was that these cellular roles seem essential for neurodegenerative diseases, given that ATXN2 depletion is surprisingly effective in protecting motor neurons and cerebellar neurons in ALS and in Spinocerebellar Ataxias (SCAs), as demonstrated in mouse models and flies.

Spinocerebellar Ataxias (SCA) are neurodegenerative disorders characterized by the degeneration of the cerebellum and its afferent and efferent connections, brainstem, spinal cord and peripheral nerves. In the past 20 years, clinical trials have been carried out in SCAs to slow down or stop disease progression and disability. However, the main findings were often negative due to the fact that the included patients had a severe degeneration of the nervous system. The best moment to start with clinical trials is before ataxia onset, where the neurodegeneration is beginning. Recently, two phases before ataxia onset were defined: the asymptomatic and the preclinical stages in SCAs. The asymptomatic phase is characterized by an absence of neurological abnormalities. However, in the preclinical phase, there are several clinical and paraclinical complaints in SCAs. “Spinocerebellar Ataxias: Early diagnosis and ethical dilemmas during preclinical trials”, was presented by Prof. Luis Velázquez-Pérez from the Center for Research and Rehabilitation of Hereditary Ataxias, Holguin, Cuba. In this talk, he commented on how the first studies of the preclinical stage were carried out in a Cuban SCA2 population. The dorsal lemniscal system, saccades movements, visual motor performance, REM sleep, and autonomic nervous system were evaluated. 35% of the preclinical subjects have cerebellar alterations such as abnormal tandem gait, mild incoordination deficit and nistagmus. The non-cerebellar manifestations were muscle cramps, sensory symptoms and sleep disorders. Clinical examination showed hyperreflexia, saccade slowing and cognitive dysfunction. The early clinical trials raise ethical dilemmas which, if not effectively addressed, may harm subjects. The high prevalence of SCA2 families in Cuba has offered great opportunities to carry intervention strategies in subjects in a preclinical state. SCA2 preclinical carriers revealed a high acceptation rate (97.5%) to be included in neurorehabilitation programs and/or clinical trials. Thus, Cuban SCA2 preclinical carriers have undergone early therapeutical approaches consisting in a rehabilitation program and an open-label clinical trial with vitamins, which improved both the subtle cerebellar manifestations and muscle cramps. Both treatments were safe in all subjects, minimizing the ethical dilemma associated with the drug side effects. Regarding the ethical concerns related to the placement of a preclinical carrier on a placebo group, all interviewed subjects that were referred agreed with these designs due to the possibility of receiving concomitant therapeutical options during the study and receiving the true drug after completion of the study, if it was effective and safe. In addition, the ethical dilemma of disease stigmatization were not observed in the treated SCA2 preclinical carriers, which could be explained by the long psychotherapeutical follow-up treatment that they received. In summary, the ethical concerns raised from the early interventions in prodromal SCA2 have led to new challenges for physicians, genetic counselors and researchers, which must be addressed via further investigations [37,38,39].

The cognitive impairment reported in 25% of patients is a common feature of Spinocerebellar Ataxia type 2 (SCA2), but has not been systematically studied, with no surrogate biomarkers having been described. In this context, Roberto Rodríguez-Labrada, also coming from the Ataxia Center in Cuba, exposed the results of a study guide to characterize new biomarkers of cognitive decline in SCA2 through clinical and electrophysiological assessments. The lecture, entitled “New biomarkers for Spinocerebellar Ataxia type 2: insight into physiopathology of cognitive decline”, included the INECO frontal screening test and an antisaccadic eye movement paradigm in 40 SCA2 patients, 37 preclinical mutation carriers and 40 healthy controls. The results evidenced a significant decrease of mean INECO scores in SCA2 patients and preclinical carriers, when compared to controls. This score was inversely correlated to expanded CAG repeats and the SARA score in the SCA2 patients, and was directly correlated to the time to ataxia onset in the preclinical carriers. Regarding the antisaccadic assessment, both disease related groups exhibited increased antisaccadic error rates and latencies, but the percentage of antisaccadic errors correction was only reduced in the patients. SCA2 patients carrying larger CAG repeats showed poorer antisaccadic performances, while preclinical carriers with shorter times to ataxia onset exhibited larger antisaccadic latencies [40,41,42]. A conclusion of this study reveals the usefulness of INECO tests and antisaccadic movements as sensitive biomarkers of executive dysfunctions in SCA2 since the early stages of the disease, as well as offering new evidence on the role of the cerebellum in cognitive functions from a human model disease.

“Metabolic mismatches and spinocerebellar Ataxia type 2 clinical phenotype”: Almaguer-Gotay Dennis from the Center for Research and Rehabilitation of Hereditary Ataxias (CIRAH) showed the results of his group, related to the characterization of the SCA2 metabolism in presymptomatic patients. They concluded that SCA2 have some metabolic mismatches beginning from the presymtomatic stages, which have a significant correlation with disease severity and progression markers [43,44]. Almaguer Mederos LE, from the same center (CIRAH), claimed that *MTHFR* C677T polymorphism is associated with disease progression in SCA 2. These results point to a possible implication of the folate metabolism in the pathophysiology of SCA2, having a role in disease progression but not in disease onset [43].

Prof. Ivón Pedroso Ibáñez delivered her lecture entitled “Erythropoietin induce neuroprotection in patients with Parkinson’s disease”. Dr. Pedroso discussed the results of a multicenter study at CIREN (International Center for Neurological Restauration), CIM (Center for Molecular Immunology) and CEMPALA (Center for Attention and Development of Animal for laboratory, Cuba), which showed that treatment strategies in Parkinson’s disease (PD) have improved patients’ quality of life without stopping their progression, and that different alternatives to modify the natural course of the disease have demonstrated neuroprotective properties of erythropoietin; particularly, Cuban recombinant form (EPOrh) and recombinant human erythropoietin with low sialic acid (NeuroEPO) have showed good results. This talk showed the results of two clinical trials for neuroprotection purposes performed by this group using patients with PD in the IV stages of the Hoenh and Yarh scale, who received an intranasal formulation of NeuroEPO. All patients were evaluated with a battery of neuropsychological scales Data showed that both molecules are tolerated by patients with Parkinson’s disease, with only mild adverse effects and a positive response to the cognitive functions. Authors considered that more studies must be carry out to underline the real relevance of this new biotechnological product. Additionally, these results contribute to the necessary screening of these types of compounds, promising protective properties to neurodegenerative diseases [45,46].

In addition, the results of an intra-nasal administration of non-hematopoietic erythropoietin promoting a clinical benefit without toxicity in spinocerebellar ataxia type 2 patients were discussed from a double-blind experiment; a placebo-controlled phase 2 clinical trial presented by Prof. Luis Velázquez and colleagues from the Center for the Research and Rehabilitation of Hereditary Ataxias, Holguín, Cuba. This study demonstrated that NeuroEpo is safe, feasible, and it suggested that it might represent a new therapeutic strategy for SCA and other neurodegenerative diseases [46].

Currently the biotechnological industry offers novel potential molecules with neuroprotective properties. An area of the meeting was reserved for the presentations of Prof. Diogo O Souza, from UFRGS, Brasil, who showed the results of his group on the neuroprotective effect of guanosine in the experimental models of brain diseases. Glutamate is the main excitatory neurotransmitter in mammalian CNS, essential for most brain activities. However, the hyperactive activation of the glutamatergic system may be potentially neurotoxic, involving the pathogenesis of various acute and chronic brain injuries. Increased glutamate causes the sustained entry of calcium and its excessive accumulation inside the cell, firing various intracellular processes that eventually lead to cell death. This increase of calcium generates the formation of free radicals that promote lipid peroxidation at the level of the membranes and the synthesis of nitric oxide, which acts as feedback and powers the excitotoxic effect by increasing the release of glutamate.

ALS is an age-related neurodegenerative disorder that is believed to have complex genetic and environmental influences in pathogenesis, but the etiologies are unidentified for most patients. Until the major causes are better defined, drug development is directed at downstream pathophysiological mechanisms, which are themselves incompletely understood. For nearly 30 years, glutamate-induced excitotoxicity has lain at the core of theories behind the spiraling events, including mitochondrial dysfunction, oxidative stress, and protein aggregation, that lead to neurodegenerative cell death in ALS [47]. The main process responsible for maintaining the extracellular glutamate levels below the toxic concentration is the glutamate uptake exerted by glutamate transporters located in neural cell membranes, mainly in astrocytes. This group showed strong evidence that the guanine-based purinergic system is effectively neuroprotective against glutamate toxicity, in acute and chronic animal models from both in vitro and in vivo studies. These results indicate that the neuroprotective guanine-based purine is the nucleoside guanosine (Guo). In vivo studies showed that Guo (i.c.v., i.p. or orally administered) protects against seizures (induced by quinolinic acid), brain ischemia and hepatic encephalopathy. The group searched for mechanisms implicated in this neuroprotection and demonstrated that this compound stimulates the astrocytic glutamate uptake in astrocyte cultures, additionally exerting neuroprotective effects that avoid the decrease in glutamate uptake [23,24,48,49].

Other molecules with neuroprotective effects were exposed by Wong-Guerra Maylin from the Center for the Development and Research of Medications, Cuba, with her paper entitled “Neuroprotective effects of JM-20 on aluminium-chloride-induced learning impairments, mitochondrial dysfunction and neuronal death in adult rats”. JM-20 is a hybrid benzodiazepine dihydropyridine molecule, with a neuroprotective effect demonstrated in brain disorders involving excitotoxicity, oxidative damage, inflammatory response and mitochondrial dysfunction. This paper showed that the treatment with JM-20 prevented the increase of the expression of pro-apoptotic signaling proteins, protecting neuronal loss and presumably cognitive impairment, and in this line of findings the authors suggested that JM-20 may prevent memory impairment via mitochondrial protection and the inhibition of the intrinsic apoptosis pathway, also suggesting a therapeutic benefit to neurodegenerative dementia like Alzheimer’s disease. The results on the regional effects of transient cerebral ischemia on astrocytes reactivity and the PI3K/Akt survival pathway, and the role of JM-20, were shown by Ramírez-Sánchez Jeney [50,51].

A Cuban experiment on a novel tool of intervention, “Transcraneal Magnetic Stimulation”, was presented by Prof. Lázaro Gómez’s lecture, “Effects of Non-Invasive Brain Stimulation in children with Autism Spectrum Disorder: a short term outcome study”.

From his approach, the core symptoms of ASD may improve with pharmacological and non-pharmacological interventions; but deficits in social interaction and language remain throughout the lifespan. Following this criteria, the study showed how non-invasive brain stimulation (NIBS) could be a valuable adjuvant therapy for ASD, from the evaluation of tolerability, safety and the clinical effect of NIBS in children with ASD. The Autism Diagnostic Interview Revisited (ADI-R), Autism Treatment Evaluation Checklist (ATEC) and the Autism Behaviour Checklist (ABC) were used to assess the intervention effects, comparing the scores before and one week, one month, three months and six months after completing all the sessions. The resting state EEG was based in the analysis of functional connectivity, which showed clear changes after treatment. A conclusion of this lecture was that both methods, tDCS and rTMS, were tolerable and safe for the patients included in the study, and that a remarkable clinical improvement in their autistic symptoms was found after treatment [52].

## 3. Conclusions

The prevalence of inflammatory processes became a major component in neurological disease, at time when a main focus in neuroscience is to look for both a major understanding of neuroimmune pathology in the context of these diseases, as well as more effective and earlier ways of intervention. Following this viewpoint, this meeting consistently highlighted the discussion of molecular mechanisms and therapeutic approaches related to a more effective neuromodulation in neurological diseases, with particular relevance to neurodevelopment and neurodegenerative diseases. Results on The TMS and NeuroEpo offered a good perspective on neuroprotectors and modulators of neuroplasticity, since the C-PC and PCB were shown to be useful and promising therapeutic options for MS, based in the remyelinating effect observed in EAE models. The microbiome was treated as a third player in MS pathogenesis. Single-target approaches are insufficient for these multifactorial diseases, and molecules are screened for different relevant targets and effects. As we observed before, natural products continue to be an important source of drugs with beneficial effects for NDs; the roles of oxidative stress and mitochondrial performance are strongly linked to AD pathogenesis; in silico research is the leading approach in drug discovery and it is being widely used, mainly for AD. The different methodologies and lines of research discussed in the symposium suggest new possible therapeutic options and provide further insights on NDs mechanisms, from basic to translational research, drug discovery (mainly from natural sources), and the use of high-technology methods. The hallmarks of the symposium were all focused on NDs and neurodevelopment disorders. The congress provided a framework for translational research to advance toward new promising neural disease-modifying therapies.
